# Feather degradation potential of *Stenotrophomonasmaltophilia* KB13 and feather protein hydrolysate (FPH) mediated reduction of hexavalent chromium

**DOI:** 10.1007/s13205-016-0370-5

**Published:** 2016-02-02

**Authors:** Khushboo Bhange, Venkatesh Chaturvedi, Renu Bhatt

**Affiliations:** 1 Department of Biotechnology, Guru Ghasidas Vishwavidyalaya (A Central University), Bilaspur, Chhattisgarh 495009 India; 2 School of Biotechnology, Banaras Hindu University, Varanasi, U.P. India

**Keywords:** *Stenotrophomonas maltophilia* KB13, Feather degradation, Feather protein hydrolysate, Cr(VI) reduction

## Abstract

An efficient keratinolytic strain of *Stenorophomonas maltophilia* KB13 was isolated from feather disposal site of Bilaspur, Chhattisgarh, India. The strain could metabolize 10 g/l chicken feathers as sole source of carbon and nitrogen. Soluble protein, amino acid, and cysteine content were found to be maximum (690.6 ± 8.7, 688.9 ± 9.12 and 21 ± 0.36 µg/ml, respectively) at late logarithmic phase of growth. Protease and keratinase activity reached its maximum level (103.26 ± 7.09 and 178.5 ± 9.10 U/ml) at the 4th day of incubation. The feather protein hydrolysate (FPH) obtained after degradation of chicken feathers was utilized to reduce hexavalent chromium. About 78.4 ± 2.4 and 63.6 ± 2.2 % reduction of 50 and 100 mg/l Cr(VI), respectively, was observed after 60 min of incubation with FPH. Further, there was no effect of autoclaved FPH on Cr(VI) reduction indicating that any bacterial enzyme was not involved in reduction process. Cr(VI) reduction was significantly inhibited by 10 mm Hg^2+^ ions indicating the role of sulfur-containing amino acids in reduction process. FTIR analysis confirmed that chromium reduction occurred due to oxidation of amino acids cysteine and cystine. This study shows that FPH arising after feather degradation can be employed as a potential candidate for the reduction of hexavalant chromium.

## Introduction

Feathers are considered as a potent waste generated in large amount by various poultry industries around the world. Feathers consist largely of insoluble protein keratin, which contains high degree of cross linking by disulfide bonds, hydrogen bonding, and hydrophobic interactions (Jeong et al. 2010). These interactions present in keratin make it highly recalcitrant in nature and its accumulation creates serious environmental concern. Various physical and chemical treatments are employed in efficient degradation of feathers, but these processes are mostly energy consuming and the resulting feather lysates are deficient in heat-sensitive amino acids such as methionine, lysine, and tryptophan (Tiwary and Gupta 2010). The hydrolysis of feathers by keratinolytic microorganisms represents alternative method to reduce energy loss and environmental pollution load (Fang et al. 2013).

Various bacteria, actinomycetes, and fungi are known to produce keratinolytic enzymes to degrade keratin and to metabolize it as carbon and nitrogen source (Tiwary and Gupta 2010). Most researches on microbial keratinases have been reported on Gram-positive bacteria, such as *Streptomyces* spp. (Tatineni et al. 2008; Syed et al. 2009) and *Bacillus* spp. (Lateef et al. 2010; Shrinivas and Naik 2011). However, some Gram-negative bacteria, such as *Chryseobacterium* sp. (Riffel et al. 2007), *Aeromonas hydrophila* K12, *Stenotrophomonas maltophia* (Yamamura et al. 2002; Jeong et al. 2010; Fang et al. 2013), *Serratia marcescens* P3 (Bach et al. 2012), and *Pseudomonas stutzeri* (Chaturvedi et al. 2014) have also been described as efficient keratin-degrading strains. All these microorganisms metabolize feathers as a source of carbon and nitrogen and release high amounts of free amino acids/short peptides in the culture medium (Yamamura et al. 2002). These are collectively termed as feather protein hydrolysates (FPH) and can be utilized as slow-release nitrogen fertilizers (Lasekan et al. 2013), antioxidants (Fontoura et al. 2005) or for production of glutathione (Taskin 2013), Exopolysaccharides (Taskin et al. 2012), Biohydrogen (Bálint et al. 2005). The presence of high amounts of free amino acids also makes it a good reducing agent. However, this property has not been much explored.

Hexavalent chromium is employed in industries such as leather tanning, metallurgy, electroplating (Ataabadi et al. 2015), petroleum refining, textile manufacturing, etc. (Allegretti et al. 2006) and, therefore, huge amounts of chromium are being discharged in the environment. Chromium exists in two oxidation states, Cr(VI) and Cr(III), where Cr(VI) is highly toxic to plants, animals, and humans. Cr(VI) has high solubility and thus is mobile in soils and ground water and whereby it gains entry into plants and animals and elicits acute toxicity. In contrast, Cr(III) precipitates as Cr(OH)_3_ under alkaline or weak acidic conditions; thus it is less mobile and toxic (Chen et al. 2007). Therefore, reduction of Cr(VI) to Cr(III) is considered as an efficient method for chromium removal and to reduce its toxicity in environment (Megharaj et al. 2003). Studies have shown that chromium can be reduced by various chemical and biological methods. Adsorption (Santhana et al. 2014), Chemicals such as ferrous [Fe(II)] compounds (Ren et al. 2013), Anion exchange resins (Deng et al. 2010), Electrolysis (Welch et al. 2005), etc. can efficiently reduce chromium compounds (Wang et al. 2012). However, these compounds are chemically synthesized, costly, and also not safe. Biological method involves use of microbes and their enzymes, which reduce chromium VI to chromium III (Thatoi et al. 2014). Several microorganisms such as *Exiguobacterium* sp. (Alam and Malik 2008), *Ochrobacterium intermedium* (Sultan and Hasnain 2007), *Stenotrophomonas* *maltophilia*, *Pantoea* sp., and *Aeromonas* sp. (Alam and Ahmad 2012) have been shown to reduce hexavalent chromium.

In the present investigation, a strain of *S. maltophia* KB13 was utilized to metabolize chicken feathers as sole source of carbon and nitrogen. FPH arising after degradation of chicken feathers by this isolate was used to reduce hexavalent chromium. This is the first report showing potential use of FPH as an inexpensive source for chromium removal.

## Materials and methods

### Materials

Feathers were procured from regional poultry farm of Bilaspur, Chhattisgarh, India, washed extensively with tap water, dried at 60 °C for 2 days, and then kept at room temperature until used. All chemicals and other reagents were of pure grade and purchased from Sigma chemicals, India.

### Preparation of media and culture condition

Bacterial strain was grown on feather meal broth (FMB) containing (g/l) K_2_HPO_4_ 0.4, KH_2_PO_4_ 0.3, NaCl 0.5, chicken feathers 10 (Tatineni et al. 2008). pH was adjusted to 7.5 before sterilization. For subculturing and storing, Feather Meal Agar (FMA) was employed, which contained (g/l) K_2_HPO_4_ 0.3, KH_2_PO_4_ 0.4, NaCl 0.5, MgCl_2_.6H_2_O 0.1, and feather 10 and agar 15. Peptone water was employed for inoculums preparation containing (g/l) peptone 10, and NaCl 5 (pH 7.4). All cultivation media were autoclaved at 121 °C, 105 kPa for 15 min.

### Isolation of feather degrading bacterial strain and phylogenetic analysis

Feather-degrading bacteria were isolated employing enrichment technique. Soil samples were collected from feather disposal site of Bilaspur, Chhattisgarh. One gram (1 g) of soil was suspended in 0.9 % saline solution and vortexed for 2–3 min intermittently. The supernatant was inoculated in 100 ml Erlenmeyer flask containing 50 ml FMB supplemented with 10 g/l chicken feathers as sole source of carbon and nitrogen. The inoculated flask was kept for incubation at 32 °C and 150 rpm in rotator shaker (Remi, CIS-24 BL) for 3 days. Five milliliter (5 ml) culture suspension was transferred subsequently to 50 ml of fresh FMB and incubated for 3 days at 32 °C. The suspension was serially diluted; 100 µl of suspension was spread on FMA and was incubated at 32 °C for 48 h. The morphologically distinct colony of bacterial cells was individually inoculated on fresh FMB and incubated at same condition mentioned above for 3 days. Feather degradation potential of each stain was assessed by estimating soluble protein content of supernatant (Bradford 1976) and by measuring residual feather content. The isolate exhibiting highest soluble protein content and least amount of residual feather was considered as potential feather degrading strain and selected for further study.

The selected strain was characterized by physiological and biochemical parameters as described in Bergey’s Manual of Determinative Bacteriology (9th edition) (Holt et al. 1994). Identification of this strain was performed by partial gene sequencing of 16S rDNA commercially (Chromous Biotech Pvt. Ltd) by primers 27 F (AGA GTT TGA TCM TGG CTC AG) and 518 R (GTA TTA CCG CGG CTG CTG G). The sequence was submitted to genbank NCBI for retrieval of accession number.

### Time course study of feather degradation

A single colony of strain KB13 was inoculated in 250 ml Erlenmeyer flask containing 50 ml of peptone water and incubated at 35 °C and 125 rpm in an orbital shaker. After overnight growth, the culture was centrifuged at 10,000 rpm for 10 min (Remi C-24 BL). The cell pellet was washed twice and suspended in 5 ml FMB (without feathers). Five percent (v/v) inoculums (3 × 10^8^ CFU ml^−1^) were added to 100 ml of FMB (containing 10 g/l chicken feather) and incubated at the same conditions. Samples (2 ml) were retrieved from each of triplicate cultures every 24 h to evaluate growth, soluble protein production, and enzyme activity. Triplicate assays were performed for each parameter.

### Estimation of growth

Growth was estimated using TTC reduction method and absorption was measured at 480 nm (De Logu et al. 2001). Briefly, to 1 ml of culture suspension 10 µl of 0.02 % TTC solution was added and was incubated at 32 °C for 30 min. The cell suspension was then centrifuged at 10,000 rpm for 10 min. Supernatant was discarded and 1 ml of 95 % ethanol was added to the cell pellet and kept for shaking (150 rpm) at 32 °C and subsequently centrifuged at 10,000 rpm for 10 min. The absorbance of supernatant was recorded at 480 nm. Protein concentration was determined using Bradford method (1976) and bovine serum albumin (BSA) as standard. The absorbance was determined spectrophotometrically at 590 nm using UV–Vis spectrophotometer (UV-1800, Shimadzu). Total amino acid content was measured by manual ninhydrin method by taking leucine as standard (Cheng et al. 2010).

### Enzyme assay

Keratinase assay was performed according to the method of Anbu et al. (2007) with minor modifications. Twenty mg (20 mg) of chopped feathers was suspended in 3.8 ml of 100 mM Tris–HCl buffer (pH 8.0) followed by addition of 200 µl of culture supernatant to it. This preparation was incubated at 50 °C and 120 rpm for 1 h and the reaction was stopped by cooling the tubes in ice chilled water. After centrifugation at 5000 rpm for 5 min, the absorbance of supernatant was measured at 280 nm. Control samples were prepared in a similar manner except that the enzyme was replaced by same volume of 100 mM Tris–HCl buffer (pH 8.0). One unit of enzyme activity was considered as the amount of enzyme required to release 1 µmol/min of tyrosine. Similarly, protease activity was determined with the above mentioned conditions using casein as the substrate.

### Estimation of cysteine content

Cysteine content was estimated by Ellman’s reagent (Ellman 1959). To 250 µl of standard cysteine solution, 2.5 ml of phosphate buffer (100 mM, pH 7.2) and 50 µl of Ellman’s reagent were added. The solution was vortexed and incubated at room temperature of 15 min. The absorbance was recorded at 412 nm. For estimation of cysteine in chicken feather hydrolysate, 250 µl of chicken feather hydrolysate obtained from (48 h old culture) was taken instead of standard cysteine solution, and the rest of steps were same as mentioned.

### Reduction of hexavalent chromium

To 1 ml of chicken feather hydrolysate, 50, 100 mg/l chromium(VI) was maintained by adding different volumes of potassium dichromate stock solution (1 mg/ml). The solution was incubated for 1 h and then residual chromium(VI) was estimated by carbazide reagent. In control, 50, 100 mg/l chromium(VI) was prepared in double-distilled water. To study the reduction of hexavalent chromium by cysteine and methionine different concentration of these amino acids (10–50 ppm) was prepared in double-distilled water and employed for chromium reduction assay. Similarly, the FPH was concentrated twice using lyophilizer (Mac HC 333, India) and was used to study chromium reduction. To study the role bacteria in reduction process, strain KB 13 was grown for 48 h in FMB (without chicken feathers) supplemented with glucose (1 g/l) and NH_4_Cl (1 g/l), respectively. After 48 h of growth the culture was centrifuged at 10,000 rpm for 10 min. The culture supernatant was filtered through Whatman filter paper No.1 and supernatant was used for reduction assay as mentioned above.

### Estimation of hexavalent chromium

Hexavalent chromium was estimated as per the method of Eaton et al. (2005). To 1 ml of potassium dichromate solution, 200 µl of 1 M sulfuric acid and 500 µl of carbazide reagent were added and incubated for 30 min. The absorbance of solution was recorded at 540 nm. In control 1 ml double-distilled water was used. To prepare carbazide reagent, to 0.05 g 1, 5 diphenyl carbazide, 2 ml glacial acetic acid was added. The final volume was maintained 25 ml by adding double-distilled water. The carbazide reagent was stored at 4 °C until use.

### Determination of minimum inhibitory concentration (MIC)

MIC was evaluated by growing the strain in nutrient broth supplemented with different concentrations of hexavalent chromium (10–50 ppm) at 32 °C for 48 h. The minimum concentration of chromium at which no growth was observed was considered as the MIC of strain.

### FTIR analysis

For FTIR analysis, potassium dichromate was treated with FPH. Final concentration was maintained at 50 mg/l. After 1 h of treatment the sample was dried in vacuum drier. Feather protein hydrolysate was taken as control. The samples were mixed with spectroscopically pure KBr in ration of 5:95 (sample: KBr). Pellets were fixed in sample holder and analysis was carried out using Shimadzu 8400 FTIR spectrometer.

## Results and discussion

Eight potential keratinolytic strains were isolated from soil samples collected from feather disposal site on the basis of enrichment technique in minimal medium containing chicken feathers (10 g/l) as sole carbon and nitrogen source. Strain KB13 was found to be the potent keratinolytic strain and only 13.8 % residual feather remained in the medium after 4 days of incubation. The strain KB13 was a Gram-negative, rod-shaped motile bacterium. 16S rDNA analysis revealed its 97 % similarity with *S. maltophilia* strain SSASC23 on BLAST search at NCBI. The strain was identified as *S. maltophilia* strain KB13 (Accession No. KC818432.1). This is in accordance with previous reports, indicating that *S. maltophilia* can metabolize chicken feathers Yamamura et al. (2002), Jeong et al. (2010) and Fang et al. (2013).

**Fig. 1:**
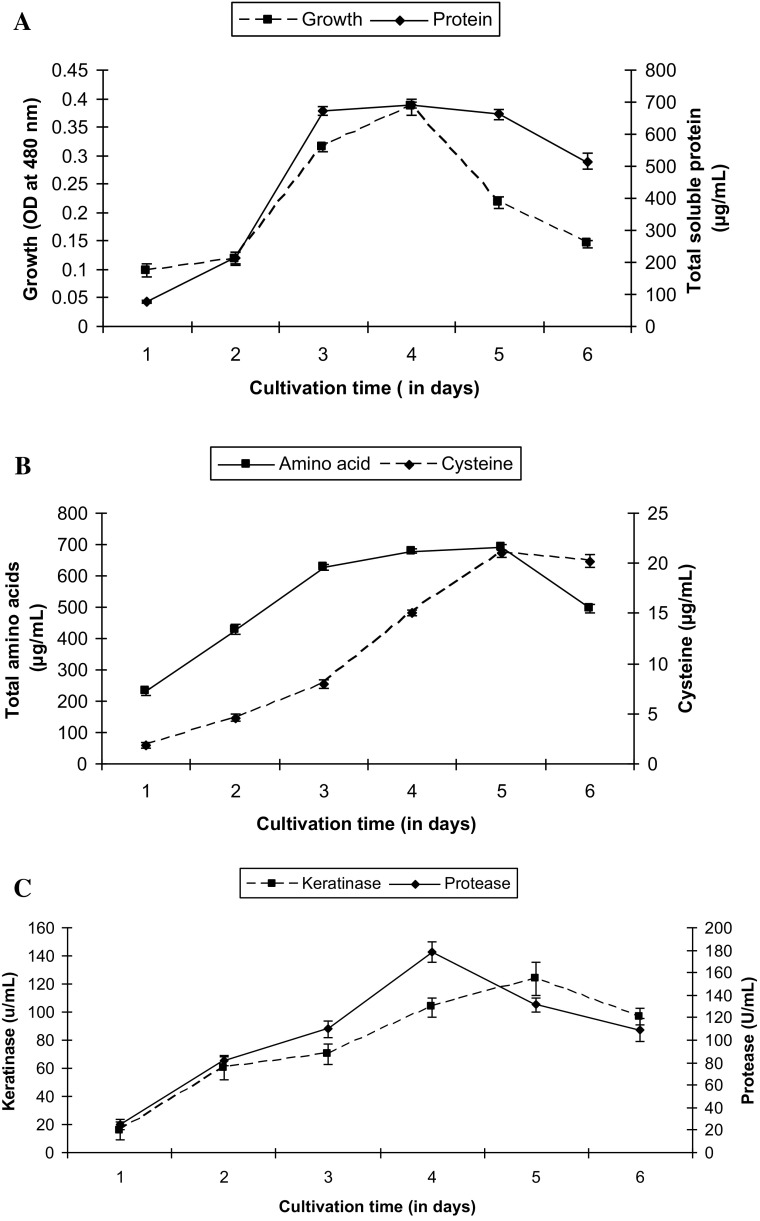
Time course study of growth and feather degradation by *S. maltophilia* KB13 on FMB supplemented with 10 g/l chicken feathers, incubated at 120 rpm, 30 °C. Time course study of **a** growth and soluble protein production, **b** amino acid and cysteine production, **c** protease and keratinase activity. The result is represented as mean ± standard error of three independent variables

The time course study of feather degradation was performed in feather meal broth containing 10 g/l feather as sole source of carbon and nitrogen. The level of soluble protein and amino acid content showed a gradual increase and reached its maximum (690.6 ± 8.7 and 688.9 ± 9.12 µg/ml) at late logarithmic phase of growth (Fig. 1a). Maximum cysteine content in FPH was 21 ± 0.36 µg/ml during this period (Fig. 1b). Feather degradation process usually starts from feather barbs and progresses with the degradation of feather rachis (Chaturvedi et al. 2014). In the present study feather degradation by the strain of *S. maltophilia* KB13 exhibited a similar trend. Keratinase and protease activity reached the maximum level (103.26 ± 7.09 and 178.5 ± 9.10 U/ml) on the 4th day of cultivation (Fig. 1c). These results were consistent with those of previous reports (Jeong et al. 2010; Chaturvedi et al. 2014).

Reports have shown that free amino acids such as cysteine, methionine, and cystine can reduce hexavalent chromium to trivalent chromium. Kwong and Pennington (1983) demonstrated that l-cysteine can reduce hexavalent chromium to trivalent chromium according to reaction.

$${\text{HCrO}}_{4}^{ - } +2{\textsc{l}}{\text{-}}\text{Cys}\;{\text{SH}} \to {\textsc{l}}{\text{-}}\text{Cystine} + {\text{HCrO}}_{3}^{ - }$$

In another study by Adari et al. (2008), it was observed that l-cystine can also reduce chromium(VI) to Chromium(III) according to the reaction.

$$3\;{\text{cystine}} + 10\;{\text{Cr}}({\text{VI}}) \to 6\;{\text{cysteic}}\;{\text{acid}} + 10\;{\text{Cr}}({\text{III}})$$

**Fig. 2:**
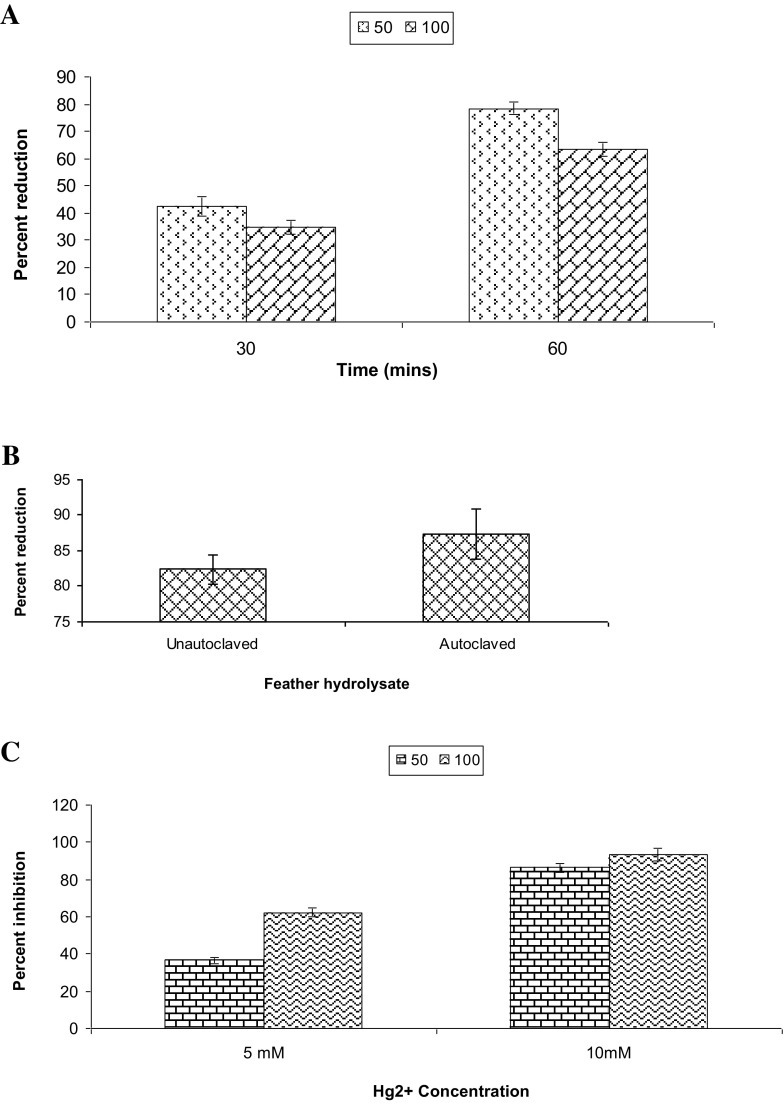
Feather protein hydrolysate mediated reduction of hexavalent chromium (**a**) reduction of 50 and 100 mg/l chromium(VI) after 30 and 60 min. **b** Reduction of 50 mg/l chromium(VI) through autoclaved and unautoclaved feather hydrolysate (**c**) reduction of 50 and 100 mg/l chromium(VI) in the presence of 5 mM and 10 mM Hg^2+^ concentration

Chicken feather hydrolysate contained high amounts of cysteine, cystine and, therefore, can be utilized for reduction of hexavalent chromium. The FPH produced at late logarithmic phase of growth after degradation of chicken feathers was employed for reduction of hexavalent chromium. In the present study, significant reduction of chromium(VI) was observed after incubation of chicken feather hydrolysate with 50 and 100 mg/l chromium(VI) for 1 h (Fig. 2a). It was also observed that the rate of reduction of 50 mg/l chromium(VI) was higher than 100 mg/l concentration. This might be due to the fact that the concentration of amino acids was limiting at higher concentration of chromium(VI). To confirm this hypothesis, FPH was concentrated twice and was employed for chromium reduction. It was observed that in the presence of FPH, reduction of hexavalent chromium was 58 ± 5.2 % and in the presence of concentrated FPH reduction was 87 ± 4.2 %. This result clearly shows that amino acids play a role in reduction of hexavalent chromium. As the amount of amino acids increases due to concentration of FPH, the rate of chromium reduction has also substantially increased. Tsopmo et al. (2014) have also previously described the reduction of hexavalent chromium by digested oat bran protein and role of cysteine in reduction process. Since, reduction of hexavalent chromium has also been reported by *S. maltophilia* (Alam and Ahmad 2012). Therefore, strain KB13 was grown for 48 h in minimal medium, the culture supernatant was used to reduce hexavalent chromium. The result showed that no reduction of chromium was observed. Further Minimum Inhibitory Concentration (MIC) of this isolate towards hexavalent chromium was calculated. MIC was found to be 40 ppm, which indicated that this strain was sensitive towards chromium. The characters of microorganisms are closely related to habitats to which they belong. *S. maltophilia* ZA-6 was recovered from soil irrigated with tannery effluents containing high amounts of chromium (Alam and Ahmad 2012) which enabled the bacterium to resist and reduce hexavalent chromium. As strain KB13 was isolated from a feather disposal site and not from the chromium-rich soil, it was able to metabolize chicken feathers but was sensitive to chromium. To further rule out the role of any bacterial enzyme in reduction process, the hydrolysate was autoclaved at 121 °C at 15 atm pressure for 20 min. It was cooled at RT and chromium oxidation assay was performed. It was observed that at a concentration of 50 mg/l chromium(VI), the rate of reduction was slightly higher in autoclaved feather hydrolysate as compared to un-autoclaved samples (Fig. 2b).

**Fig. 3:**
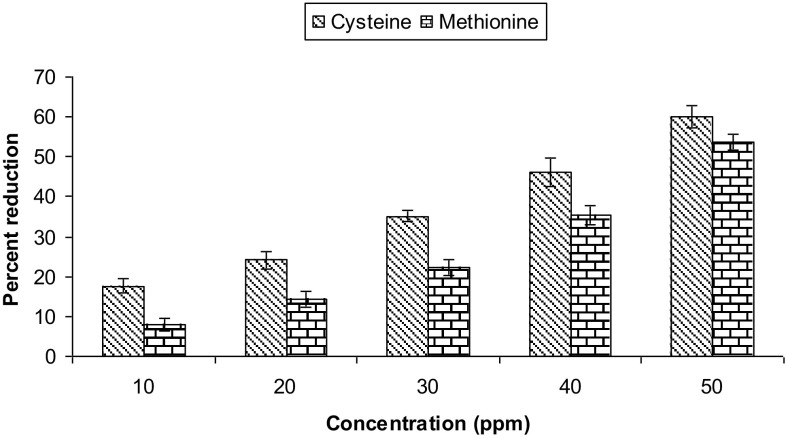
Reduction of 50 mg/l hexavalent chromium in the presence of varying concentrations of cysteine and methionine, respectively, after 1 h of incubation

Since concentration of amino acids vary considerably in FPH, reduction of 50 mg/l hexavalent chromium was performed with varying concentrations of cysteine and methionine, respectively (Fig. 3). A dose-dependent relationship was observed with chromium reduction and concentration of amino acid: with an increase in concentration of amino acid an increase in chromium reduction was observed. However, the reduction was low as compared to that observed in FPH. Since FPH contains a mixture of amino acids, reduction takes place by combined action of these amino acids and higher reduction was observed. Quievryn et al. (2001) have reported that glutathione and cysteine are main reducers of hexavalent chromium in humans exposed to high concentration of chromium. In this study, hexavalent chromium was reduced to chromium(III). We proposed that a similar mechanism is involved in reduction of hexavalent chromium by FPH. Since concentration of cysteine in FPH is very high (21 ± 0.36 µg/ml), high amount of hexavalent chromium (50 and 100 mg/l) was reduced.

To further confirm this hypothesis, chromium reduction was studied in the presence of Hg^2+^ ions, since Hg^2+^ ions are considered as potent inhibitor of sulfur-containing amino acids such as cysteine, cystine, and methionine, respectively. Hg^2+^ irreversibly binds to these amino acids and inhibits their activity (Focardi et al. 2012). To further confirm the role of these amino acids in reduction of hexavalent chromium, 5 and 10 mM concentration of Hg^2+^ ions were added to FPH and chromium reduction was estimated (Fig. 2c). It was observed that in the presence of 5 mm Hg^2+^ ions, 36.5 ± 1.4 and 62.3 ± 2.3 % inhibition in chromium reduction at 50 and 100 mg/l chromium(VI) was observed. Similarly, in the presence of 10 mM Hg^2+^ ions, 86.4 ± 2.4 and 93.2 ± 3.5 % inhibition in chromium reduction at 50 and 100 mg/l chromium(VI) was observed. This result clearly demonstrates the role of sulfur-containing amino acids in reduction of hexavalent chromium.

**Fig. 4:**
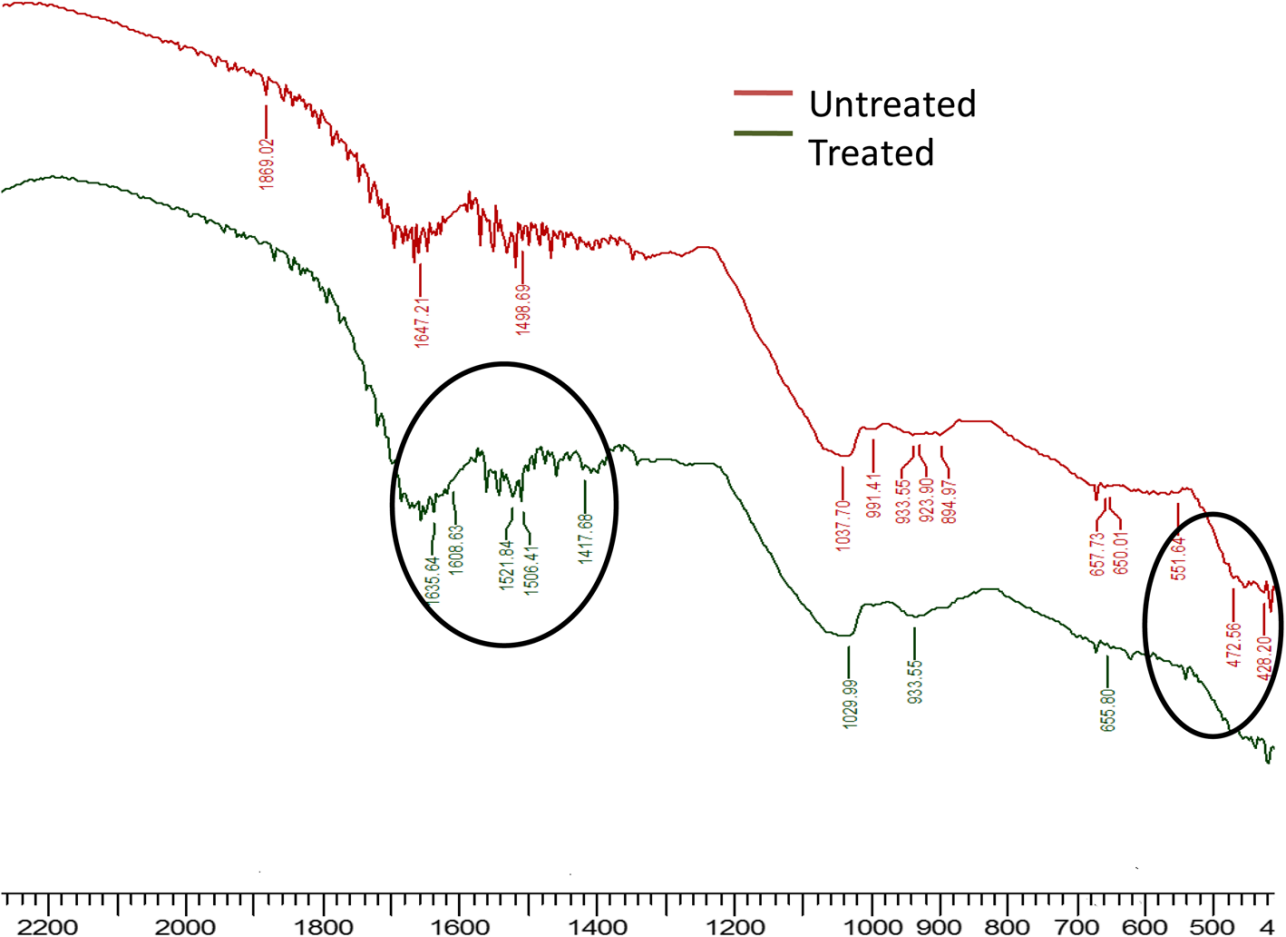
FTIR spectrum of feather protein hydrolysate (*red*) and Cr(VI) treated feather hydrolysate (*green*) presented the chemical groups involved in Cr(VI) reduction process

To further confirm the role of amino acid cysteine in reduction of hexavalent chromium, FTIR analysis of feather protein lysate and feather protein lysate containing 50 mg/l potassium dichromate was performed. The FTIR spectrum is depicted in Fig. 4. The FTIR spectrum of feather protein lysate showed peaks between 570 and 705 cm^−1^, i.e. at 472.56, 551.64 cm^−1^ corresponding to S–S stretching of disulfide bond (Lo et al. 2012). In feather hydrolysate-containing chromium, these peaks were absent and a new peaks at 1608.63, 1521.84, 1506.41, and 1029.99 cm^−1^ corresponding to S=O bond present in cysteic acid were observed. This confirms that chromium reduction is taking place due to oxidation of cysteine to cystine and then to cysteic acid. The findings of this study are consistent with previous reports (Adari et al. 2008).

## Conclusion

In this study, feather degradation by *S. maltophilia* KB13 has shown to be an efficient and ecofriendly approach to reduce the accumulation of feathers in the environment. The FPH obtained after degradation of chicken feather could be efficiently utilized for the reduction of chromium. The results of this study clearly demonstrate that sulfur-containing amino acids such as cysteine and cysteine were solely responsible for the reduction process and that no bacterial enzyme was involved. This is the first report showing the potential use of FPH as an inexpensive source for chromium removal.
